# Prognostic impact of time from diagnosis to start of radiotherapy in anal cancer

**DOI:** 10.1111/codi.70569

**Published:** 2026-08-12

**Authors:** Jakob Hallström, Anders Johnsson, Martin P. Nilsson

**Affiliations:** ^1^ Division of Oncology and Pathology, Department of Clinical Sciences Lund University Lund Sweden; ^2^ Department of Hematology, Oncology and Radiation Physics Skåne University Hospital Lund Sweden

**Keywords:** anal cancer, anal carcinoma, anus neoplasms, prognosis, radiotherapy, waiting lists

## Abstract

**Aim:**

A prolonged time from diagnosis to start of radiotherapy (TTRT) for malignant disease may negatively influence survival, but studies in squamous cell carcinoma of the anus (SCCA) are largely lacking. The purpose of this study was to investigate the impact of TTRT on overall survival (OS) and anal cancer‐specific survival (ACSS) in a nationwide population‐based cohort from the Swedish Anal Cancer Registry.

**Method:**

Patients with non‐metastatic SCCA treated with curative intent definitive radiotherapy (93.3% concomitant chemotherapy) during 2015–2023 were included (*n* = 958). According to national guidelines TTRT should be ≤ 46 days and this was used as a cut‐off in the primary analyses. Cox proportional hazards models were used to estimate hazard ratios for OS and ACSS. Variables with *p* < 0.10 in univariable analyses were entered into multivariable models, with TTRT included in all models.

**Results:**

The median TTRT was 48 days (range 17–350), and in 55% of the patients it exceeded 46 days. Old age, poor performance status, small tumour size, absence of lymph node metastasis and no concomitant chemotherapy were associated with TTRT >46 days. With a median follow‐up of 43 months, TTRT > 46 days had no impact on OS or ACSS. Sensitivity analyses using alternative TTRT cut‐offs and restricting the analysis to high‐risk subgroups showed similar results.

**Conclusion:**

Moderate delays beyond 46 days from diagnosis to radiotherapy initiation did not adversely affect OS or ACSS in patients with SCCA.

**What does this paper add to the literature?**

The findings of this study suggest that a moderate time delay between the date of diagnosis and the initiation of radiotherapy for anal cancer does not adversely affect the chance of cure.

## INTRODUCTION

Squamous cell carcinoma of the anus (SCCA) is a rare disease, with approximately 200 new cases in Sweden per year. A majority (90%) of SCCA are caused by human papilloma virus (HPV), of which immunohistochemical p16 staining is a surrogate marker. The standard treatment is radiotherapy combined with chemotherapy (chemoradiotherapy, CRT), with salvage surgery reserved for patients who do not achieve a complete response. The treatment is often effective, leading to 70%–80% long‐term survival [1]. Advanced tumour stage, male gender and HPV‐independent tumours are known factors associated with a worse prognosis [2].

Tumour progression with local growth, infiltration into surrounding tissues and metastasis occur over time. Accordingly, the waiting time from detection of cancer to initiation of treatment could be of importance. Previous reviews and meta‐analyses have suggested that a prolonged waiting time is associated with worse outcome [3, 4]. However, results have been inconsistent and seem to vary between cancer diagnoses, clinical situations (neoadjuvant, primary or adjuvant) and types of treatment (surgery, radiotherapy or chemotherapy). The impact of waiting time in SCCA has only been investigated in one previous study. Using the Surveillance, Epidemiology, and End Results (SEER) database, Abdel‐Rahman et al. found inferior survival for patients with a waiting time exceeding 2 months from diagnosis of SCCA until initiation of any type of treatment, be it radiotherapy, chemotherapy or surgery [5].

The Swedish standardized cancer pathway for SCAA (i.e., national guidelines) recommends that treatment should start within 46 days from the date of high clinical suspicion of anal cancer. The 46‐day limit reflects a consensus‐based estimate of the time required to complete essential diagnostic procedures, including imaging, multidisciplinary evaluation and treatment planning in routine clinical practice [1]. The main purpose of the present study was to investigate the impact of waiting time on survival outcomes in a large cohort of patients with SCCA receiving radiotherapy in a standardized setting, based on the Swedish Anal Cancer Registry (SACR). Another purpose of this study was to explore what baseline characteristics were associated with waiting time.

## METHOD

### Study design and population

This was a nationwide, retrospective, population‐based study. Data for the variables presented in Table 1 were retrieved from the SACR. Patients with non‐metastatic SCCA treated with curative intent radiotherapy (defined as ≥ 50 Gy) during the years 2015–2023 were included. Exclusion criteria were missing follow‐up data, surgical excision prior to radiotherapy and the use of neoadjuvant chemotherapy.

**TABLE 1:** Patient and treatment characteristics.

	All patients	TTRT		
	*n* (%)	≤ 46 days, *n* (%)	> 46 days, *n* (%)	*P*
Total *n*. of patients	958 (100)	427 (100)	531 (100)	
Gender				
Female	738 (77.0)	334 (78.2)	404 (76.1)	0.43
Male	220 (23.0)	93 (21.8)	127 (23.9)	
Median age, years (range)	67.0 (29–94)	66.3 (33–93)	68.8 (29–94)	**< 0.001**
WHO performance status				
0	656 (72.1)	309 (75.4)	347 (69.4)	**0.046**
1–3	254 (27.9)	101 (24.6)	153 (30.6)	
Missing	48	17	31	
Primary tumour size, mm				
Median (range)	44 (1–150)	45 (5–120)	40 (1–150)	**0.003**
Primary tumour size ≥ 40 mm	546 (60.0)	267 (64.2)	279 (56.5)	**0.02**
Primary tumour size ≥ 70 mm	155 (17.0)	80 (19.2)	75 (15.2)	0.10
Missing	48	11	37	
T stage				
1	117 (12.3)	33 (7.7)	84 (16.0)	**< 0.001**
2	393 (41.3)	175 (41.0)	218 (41.6)	
3	233 (24.5)	118 (27.6)	115 (22.0)	
4	208 (21.9)	101 (23.7)	107 (20.4)	
Missing	7	0	7	
Lymph node metastasis				
Yes	459 (49.9)	232 (57.3)	227 (44.1)	**< 0.001**
No	461 (50.1)	173 (42.7)	288 (55.9)	
Missing	38	22	16	
p16 status				
Positive	765 (91.2)	352 (91.4)	413 (91.0)	0.82
Negative	74 (8.8)	33 (8.6)	41 (9.0)	
Missing	119	42	77	
Radiotherapy dose, Gy; median (range)				
Primary tumour	57.5 (51–66)	57.5 (51–64)	57.5 (51–66)	0.08
Lymph node metastasis	50.5 (46–64)	50.5 (46–64)	50.5 (46–64)	0.54
Elective lymph nodes	41.6 (18‐60)^a^	41.6 (34–55)	41.6 (18–60)	0.47
Chemotherapy				
No chemotherapy	64 (6.7)	19 (4.4)	45 (8.5)	**0.02**
Monotherapy	14 (1.5)	4 (0.9)	10 (1.9)	
Combination therapy	880 (91.9)	404 (94.6)	476 (89.6)	
Stoma before start of radiotherapy	126 (13.2)	52 (12.2)	74 (13.9)	0.42

*Note*: Bold indicates *p* values < 0.005.

Abbreviation: TTRT, time from diagnosis to start of radiotherapy.

^a^ The wide range of reported radiotherapy doses to elective areas was driven by a small number of outliers.

The SACR was initiated in 2015 and now has a near‐complete (95%) coverage since the national centralization of anal cancer care in 2017–2019. Since then, definitive CRT has been delivered exclusively at four university hospitals (Umeå, Uppsala, Gothenburg and Lund). All patients are discussed at a national multidisciplinary tumour board before treatment initiation, with oncologists, colorectal surgeons and radiologists present. Registration to SACR is web‐based and performed prospectively at each site, including patient demographics, tumour characteristics, treatment details and outcomes. Patients can actively opt out from being included in SACR, but very few patients do. Since information on the date of high clinical suspicion was lacking for many patients in SACR, date of diagnosis was used instead in the analyses. To test whether this was appropriate, the first step was to investigate the correlation between date of high clinical suspicion and date of diagnosis, in cases where both dates were available in SACR. A strong correlation was observed (Kendall's tau‐b correlation: 0.985, *p* = < 0.001), supporting the use of date of diagnosis as an appropriate proxy variable. In the following, only date of diagnosis is used.

In Sweden, definitive CRT for anal cancer is currently stratified by tumour stage and nodal status into two radiotherapy dose schedules, based on the first national guidelines, broadly implemented in 2017. Patients with early tumours (T1‐2 < 4 cm, N0) receive 54 Gy to the primary tumour and 41.6 Gy to elective lymph nodes in 27 fractions, with 1 cycle of mitomycin and 5‐fluorouracil or capecitabine. Patients with locally advanced tumours (T2 ≥ 4 cm, or T3–4, or N+) typically receive 57.5 Gy to the primary tumour, 50.5 Gy to lymph node metastases and 41.6 Gy to elective lymph nodes in 27 fractions, with 2 cycles of mitomycin and 5‐fluorouracil or capecitabine. Prior to the national coordination in 2017, similar dosing schedules as above were used, but with slightly more variation between treatment centres.

### Definitions and endpoints

Time to radiotherapy (TTRT) was defined as the number of days from diagnosis to the first day of radiotherapy. Date of diagnosis was defined as the date of biopsy, not the date of the pathological report confirming SCCA. The primary aim of the study was to investigate the impact of TTRT on overall survival (OS) and anal cancer‐specific survival (ACSS). OS was defined as time from diagnosis to death from any cause. ACSS was defined as time from diagnosis to death occurring after persistent or recurrent SCCA. Based on the national guidelines, patients were divided into two groups: TTRT ≤ 46 days and TTRT > 46 days. Pre‐planned sensitivity analyses were performed to investigate the impact of TTRT on prognosis in subgroups of patients with high‐risk characteristics, further detailed in the results section.

### Statistical analysis

Correlation between dates was carried out using Kendall's tau‐b and visualized by scatterplot. Baseline characteristics were compared between TTRT groups using chi‐square or Fisher's exact tests for categorical variables, and *t*‐tests or Mann–Whitney *U* for continuous variables, as appropriate. Cox proportional hazards models estimated hazard ratios (HRs) with 95% confidence intervals (CIs) for OS and ACSS. Variables with *p* < 0.10 in univariable models were entered into multivariable models. In addition, TTRT was included in all multivariable models. Survival probabilities were calculated with the Kaplan–Meier method and compared with the log‐rank test. All significance tests were two‐sided, and *p* values < 0.05 were considered significant. Statistical analysis was conducted using IBM SPSS statistics v. 28.0.1.0.

## RESULTS

### Study population

A total of 958 non‐metastatic SCCA patients, all treated with curative intent RT, met the inclusion criteria and constituted the present study population. The median age was 67 years and 77% were female. Almost all (98%) were staged prior to treatment with both magnetic resonance imaging (MRI) and [18F]‐fluorodeoxyglucose positron emission tomography with computed tomography (PET‐CT), and 13% had a pre‐treatment diverting stoma. Baseline patient and treatment characteristics are listed in Table 1.

### Factors associated with the time from diagnosis to start of radiotherapy

**FIGURE 1:**
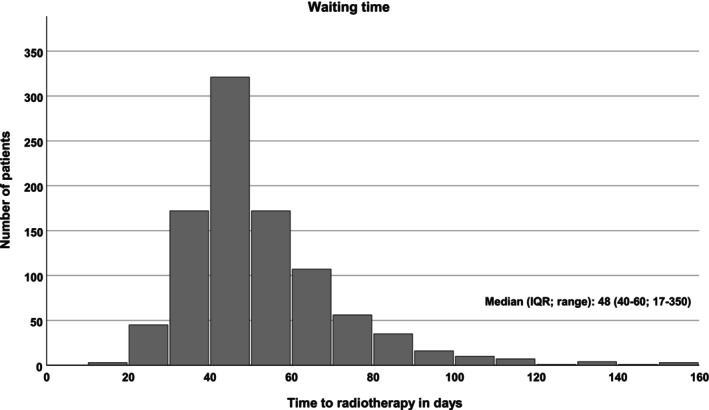
Waiting time distribution.

The median TTRT was 48 days (interquartile range 40–60, total range 17–350) (Figure 1). In 55% of the patients, TTRT exceeded 46 days. Older age, performance status ≥ 1, smaller primary tumour size, absence of lymph node metastasis and no concomitant chemotherapy were associated with TTRT > 46 days. Gender, p16‐status, and diverting stoma before treatment initiation did not affect TTRT (Table 1).

### Survival outcomes

Median follow‐up time was 43 months. At the end of follow‐up 154 patients had died, 86 of whom from SCAA. In multivariable analysis, performance status ≥ 1, larger tumour size, p16 negativity and omission of concomitant chemotherapy were associated with both inferior OS and inferior ACSS. Older age was associated with inferior OS but not with ACSS (Tables 2 and 3).

**TABLE 2:** Prognostic factors for overall survival.

Variable	Overall survival (OS)			
	Univariate		Multivariate	
	HR (95% CI)	*P*	HR (95% CI)	*P*
Age at diagnosis (years)	1.04 (1.03–1.06)	**< 0.001**	1.03 (1.01–1.06)	**0.002**
Male gender	1.47 (1.03–2.08)	**0.03**	1.08 (0.66–1.78)	0.75
Performance status ≥ 1	3.29 (2.37–4.56)	**< 0.001**	2.44 (1.59–3.73)	**< 0.001**
Primary tumour size (cm)	1.22 (1.15–1.30)	**< 0.001**	1.22 (1.11–1.34)	**< 0.001**
T4	1.58 (1.11–2.23)	**0.01**	1.11 (0.70–1.76)	0.65
Lymph node metastasis	1.51 (1.08–2.09)	**0.02**	1.20 (0.77–1.86)	0.42
p16 negative	2.91 (1.85–4.59)	**< 0.001**	3.22 (1.85–5.61)	**< 0.001**
No concomitant chemotherapy	2.60 (1.69–3.99)	**< 0.001**	2.07 (1.12–3.80)	**0.02**
TTRT > 46 days	1.05 (0.76–1.45)	0.77	0.82 (0.54–1.22)	0.32

*Note*: Bold indicates *p* values < 0.005.

Abbreviations: HR, hazard ratio; TTRT, time from diagnosis to start of radiotherapy.

**TABLE 3:** Prognostic factors for anal cancer‐specific survival.

Variable	Anal cancer specific survival (ACSS)			
	Univariate		Multivariate	
	HR (95% CI)	*P*	HR (95% CI)	*P*
Age at diagnosis (years)	1.02 (1.00–1.04)	**0.04**	1.01 (0.98–1.03)	0.71
Male gender	2.25 (1.46–3.48)	**< 0.001**	1.11 (0.59–2.07)	0.75
Performance status ≥ 1	3.17 (2.05–4.89)	**< 0.001**	2.21 (1.29–3.79)	**0.004**
Primary tumour size (cm)	1.32 (1.22–1.42)	**< 0.001**	1.31 (1.17–1.46)	**< 0.001**
T4	1.81 (1.15–2.84)	**0.01**	1.23 (0.69–2.19)	0.49
Lymph node metastasis	2.60 (1.60–4.22)	**< 0.001**	1.76 (0.95–3.26)	0.07
p16 negative	4.63 (2.80–7.67)	**< 0.001**	5.95 (3.10–11.42)	**< 0.001**
No concomitant chemotherapy	2.10 (1.11–3.95)	**0.02**	3.07 (1.36–6.97)	**0.01**
TTRT > 46 days	0.81 (0.53–1.24)	0.33	0.72 (0.43–1.21)	0.22

*Note*: Bold indicates *p* values < 0.005.

Abbreviations: HR, hazard ratio; TTRT, time from diagnosis to start of radiotherapy.

### Impact of TTRT on survival

**FIGURE 2:**
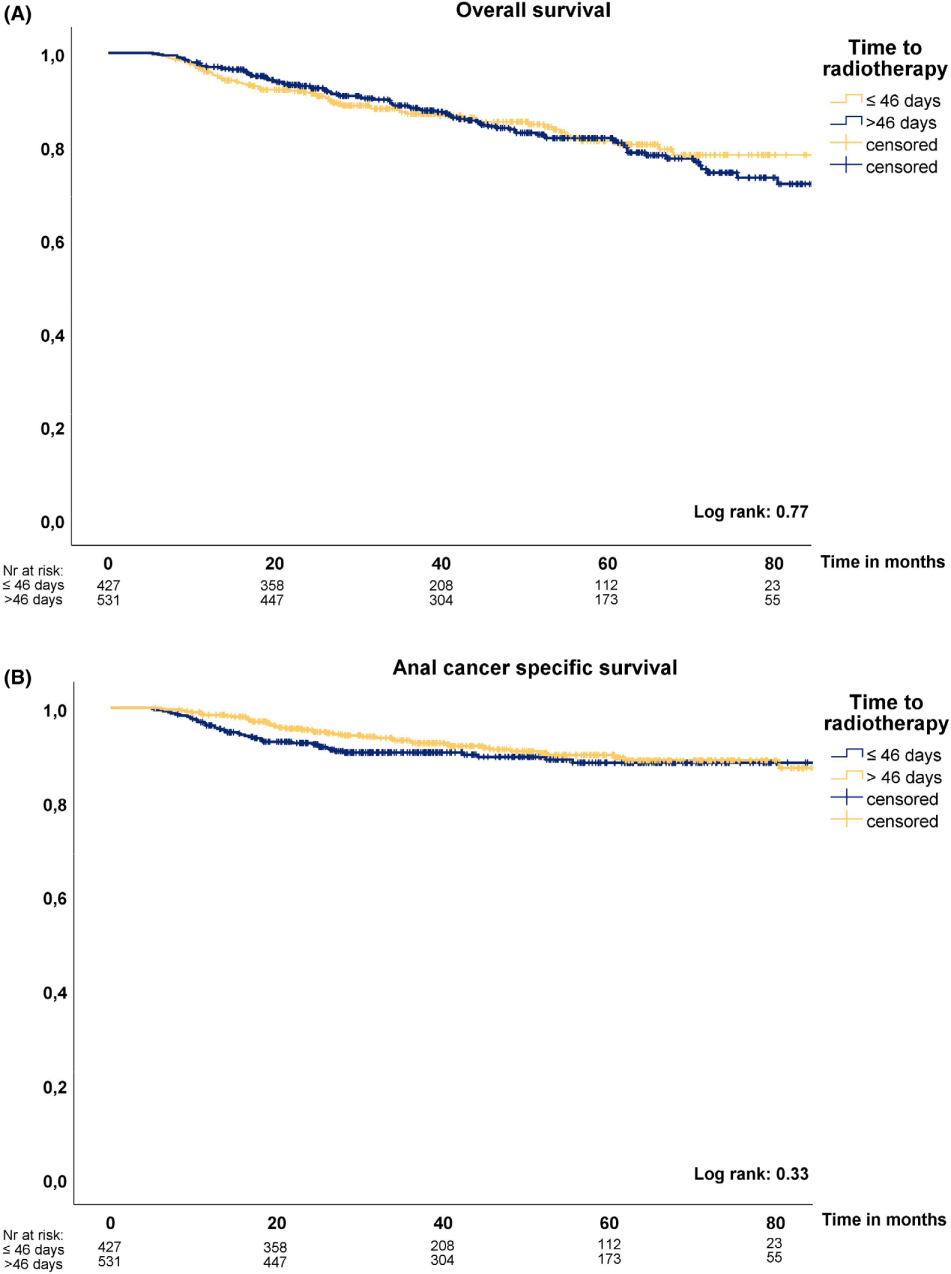
Overall survival (A) and anal‐specific survival (B) by time from diagnosis to start of radiotherapy ≤ 46 days or > 46 days.

TTRT > 46 days had no impact on OS or ACSS in univariable or multivariable analysis (all *p* > 0.20) (Tables 2 and 3). Neither did the Kaplan–Meier curves show any significant differences in OS or ACSS between TTRT ≤ 46 days or > 46 days (Figure 2). Post hoc analyses of recurrence‐free survival, presented in Table S1, did not demonstrate any association with TTRT, consistent with our findings for OS and ACSS.

Pre‐planned sensitivity analyses were performed; first, by changing the TTRT cut‐off to the upper quartile (60 days), and second, by restricting the analyses to high‐risk patients based on primary tumour size (> 40 mm and > 70 mm, respectively) and the presence of lymph node metastasis. None of these analyses showed any associations between TTRT and OS or ACSS (Table 4).

**TABLE 4:** Sensitivity analyses, univariate.

	OS		ACSS	
	HR	*P*	HR	*P*
TTRT ≥ 60 days, all patients	1.08	0.65	0.89	0.64
TTRT > 46 days, restricted to patients with primary tumour size ≥ 40 mm	1.03	0.86	0.74	0.22
TTRT > 46 days, restricted to patients with primary tumour size ≥ 70 mm	1.02	0.95	0.74	0.42
TTRT > 46 days, restricted to patients with lymph node metastasis	1.07	0.76	1.06	0.84

Abbreviations: ACSS, anal cancer‐specific survival; HR, hazard ratio; OS, overall survival; TTRT, time from diagnosis to start of radiotherapy.

## DISCUSSION AND CONCLUSIONS

In this large, nationwide cohort of patients with SCCA, a moderately prolonged time from diagnosis to start of radiotherapy did not affect OS or ACSS.

The prognostic impact of waiting time to start of treatment has been subject of many previous publications in a wide variety of malignancies and treatments; some have shown worse outcome with delayed treatment [6, 7, 8, 9, 10], whereas others have not [11, 12, 13, 14]. In anal cancer there is, to the best of our knowledge, only one previous study on this subject. Abdel‐Rahman et al. [5] analysed patients from the SEER database and found inferior OS and ASCC with waiting time longer than 2 months from diagnosis to start of treatment. The authors recognized some limitations in their study, including lack of completeness of the variables, incomplete information on given treatments and poor precision for the time calculations since dates were not available in the SEER database, only months of diagnosis and treatment. Whether these factors can explain the discrepancies between their and our findings is unclear. The SEER database includes patients treated in the United States, where healthcare is generally privately funded. In such a system, it is likely that patients with longer waiting times differ from patients with shorter waiting times regarding unmeasured patient‐ and system‐related factors. These may include comorbidity, socioeconomic status and access to care, which can also affect outcomes. The publicly funded health care in Sweden makes our study population well suited for the question of prognostic impact of waiting time. We used a 46 days waiting time cut‐off in the primary analyses, based on the Swedish guidelines, but a sensitivity analysis using a later cut‐off of 60 days was also performed, again showing no negative effect on OS or ASCC, not even in large tumours.

The observed waiting times likely reflect real‐world clinical practice, including diagnostic procedures, multidisciplinary tumour board discussions, radiotherapy planning and, in some cases, additional clinical interventions such as stoma formation or optimization of patient condition. Patients with a prolonged waiting time were older and had worse performance status. Despite not being explicitly supported by healthcare policies in Sweden, the results suggest that younger and otherwise healthy cancer patients were prioritized over older cancer patients. Furthermore, patients with TTRT longer than 46 days had less advanced tumours (lower T and N stage) and were less prone to receive concomitant chemotherapy (Table 1). This probably reflects that patients with more advanced tumours were handled with higher priority in the daily decision process, which is the reason why smaller tumour size and N0 status were associated with longer TTRT. From a clinical perspective, this represents a reasonable and appropriate prioritization. However, it cannot be excluded that this circumstance may have contributed to the lack of prognostic impact of waiting time in the present study. Conversely, there was no tendency for worse survival with TTRT >46 days in the multivariable analysis after correction for tumour stage, which strengthens the main conclusion of the study.

The relatively narrow distribution of TTRT in our cohort, with few patients treated at earlier time points, may have limited the ability to detect an association between waiting time and outcomes. In other malignancies, for example, head and neck cancer, treatment delays have been associated with worse outcomes, particularly at shorter time intervals [15]. This suggests that the potential effects of waiting time may be more evident when comparing earlier versus delayed treatment initiation, which was limited in our cohort. The absence of an observed association between waiting time and survival in our study may also have other explanations. First, the biology of HPV‐related SCCA, which constituted the majority of our cohort, may be more radiosensitive and less prone to rapid progression during short delays as studies of HPV‐associated head neck cancer suggest [16, 17]. Second, treatment was standardized across four specialized centres with national guidelines and multidisciplinary tumour boards, ensuring uniform care and minimizing variation in outcomes attributable to centre differences [1].

Independent predictors of survival in our study were consistent with established prognostic factors in SCCA. Poor performance status, larger tumour size, p16 negativity and absence of concomitant chemotherapy were strongly associated with worse OS and ACSS, in line with prior reports [1, 2]. Similar prognostic patterns have been described in recent population‐based studies [18, 19], supporting the validity of our findings. This indicates good representativeness, which should strengthen the validity of our findings. Other strengths of the present study include the large sample size, high national coverage of the SACR and standardized treatment practices after centralization of care.

Limitations of the study include its retrospective nature, potential residual confounding, from for example, lifestyle and socioeconomic factors, that are not registered in SACR. Another possible problem, as with all national quality registries run by the health authorities, is the lack of regular monitoring of all data entries. No validation studies of SACR have been performed yet and the frequency of errors is unknown. It is inevitable that large databases contain errors, which must be acknowledged in the interpretation of data. A validation study of the Swedish Colorectal Cancer Registry, which is another national registry that is similar to SACR in design, showed high agreement with re‐abstracted data from medical records for cancer recurrence (95.7%) and histopathology (93.4%), but a lower rate (84.3%) for postoperative complications [20]. Finally, a small degree of immortal time bias cannot be excluded in our study, as patients with delayed radiotherapy could only be included in the study cohort if they ultimately proceeded to curative intent radiotherapy.

We point out that the lack of prognostic impact of waiting time in our study should not be used as an argument for delaying the start of radiotherapy. There are certainly other reasons for starting radiotherapy as early as possible, not least to ease the psychological burden on the patient and to start reducing the severe symptoms that SCCA often cause. In case of rapidly growing tumours, earlier initiation of radiotherapy may also allow for smaller radiation fields and potentially reduced toxicity.

In conclusion, the results of this study suggest that moderate delays beyond 46 days from diagnosis to radiotherapy initiation do not adversely affect survival outcomes in patients with SCCA. Even though redundant delays should be avoided, our findings suggest that if the clinical situation calls for further measures, such as placement of a diverting stoma or complementary radiological examinations, these can be performed without compromising the prognosis.

## AUTHOR CONTRIBUTIONS

**Anders Johnsson:** Conceptualization; formal analysis; methodology; project administration; writing – review and editing. **Martin P. Nilsson:** Conceptualization; formal analysis; writing – review and editing; project administration; funding acquisition; methodology. **Jakob Hallström:** Conceptualization; methodology; data curation; formal analysis; writing – original draft; writing – review and editing.

## FUNDING INFORMATION

The work was funded by Mrs. Berta Kamprad's Cancer Foundation (grant number 2022‐3‐363) and Skane County Consil's Research Foundation.

## CONFLICT OF INTEREST STATEMENT

AJ has received lecture honoraria from Insyte. The other authors declare no conflict of interest.

## ETHICS STATEMENT

The study was approved by the Swedish Ethical Review Authority (Dnr 2020/06085).

## CONSENT

The need for informed consent was waived given the retrospective nature of the study.

## Supporting information


**Table S1.** Univariable and Multivariable Cox Regression of RFS.

## Data Availability

The data that support the findings of this study are available on request from the corresponding author. The data are not publicly available due to privacy or ethical restrictions.
