# Supplementary material for: Prognostic impact of time from diagnosis to start of radiotherapy in anal cancer
Source: Colorectal Dis. 2026 Aug 12;28(8):e70569. doi: 10.1111/codi.70569 (PMC13469718; doi:10.1111/codi.70569)
Supplement: Supplementary file 1 [file CODI-28-0-s001.pdf]

Supplementary Table 1. Univariable and Multivariable Cox Regression of RFS

Variable	**Recurrence-free survival (RFS)**			
	Univariable		Multivariable	
	HR (95% CI)	*P*	HR (95% CI)	*P*
Age at diagnosis (years)	1.01 (1.00-1.03)	0.21		
Male gender	**1.81 (1.28-2.55)**	**<0.001**	1.17 (0.73-1.87)	0.51
Performance status ≥ 1	**2.58 (1.85-3.61)**	**<0.001**	**2.11 (1.41-3.15)**	**<0.001**
Primary tumor size (cm)	**1.30 (1.22-1.38)**	**<0.001**	**1.29 (1.18-1.41)**	**<0.001**
T4	**1.59 (1.12-2.26)**	**0.01**	0.87 (0.54-1.39)	0.55
Lymph node metastasis	**2.11 (1.48-3.00)**	**<0.001**	1.46 (0.92-2.33)	0.11
p16 negative	**4.48 (2.97-6.77)**	**<0.001**	**5.06 (3.03-8.44)**	**<0.001**
No concomitant chemotherapy	1.51 (0.87-2.63)	0.15		
TTRT > 46 days	0.84 (0.61-1.16)	0.29	0.82 (0.55-1.22)	0.34

Abbreviations: HR, hazard ratio; TTRT, time from diagnosis to start of radiotherapy
